# Genetic analysis of invasive *Escherichia coli* in Scotland reveals determinants of healthcare-associated versus community-acquired infections

**DOI:** 10.1099/mgen.0.000190

**Published:** 2018-06-22

**Authors:** Cosmika Goswami, Stephen Fox, Matthew Holden, Martin Connor, Alistair Leanord, Thomas J. Evans

**Affiliations:** ^1^​ Institute of Infection, Immunity and Inflammation, University of Glasgow, Glasgow, UK; ^2^​ University of St Andrews, UK; ^3^​ Dumfries and Galloway Royal Infirmary, UK

**Keywords:** bacteraemia, antibiotic resistance, genome sequencing, genome-wide association study

## Abstract

Bacteraemia caused by *Escherichia coli* is a growing problem with a significant mortality. The factors that influence the acquisition and outcome of these infections are not clear. Here, we have linked detailed genetic data from the whole-genome sequencing of 162 bacteraemic isolates collected in Scotland, UK, in 2013–2015, with clinical data in order to delineate bacterial and host factors that influence the acquisition in hospital or the community, outcome and antibiotic resistance. We identified four major sequence types (STs) in these isolates: ST131, ST69, ST73 and ST95. Nearly 50 % of the bacteraemic isolates had a urinary origin. ST69 was genetically distinct from the other STs, with significantly less sharing of accessory genes and with a distinct plasmid population. Virulence genes were widespread and diversely distributed between the dominant STs. ST131 was significantly associated with hospital-associated infections (HAIs), and ST69 with those from the community. However, there was no association of ST with outcome, although patients with HAI had a higher immediate mortality compared to those with community-associated infections (CAIs). Genome-wide association studies revealed genes involved in antibiotic persistence as significantly associated with HAIs and those encoding elements of a type VI secretion system with CAIs. Antibiotic resistance was common, and there were networks of correlated resistance genes and phenotypic antibiotic resistance. This study has revealed the complex interactions between the genotype of *E. coli* and its ability to cause bacteraemia, and some of the determinants influencing hospital or community acquisition. In part, these are shaped by antibiotic usage, but strain-specific factors are also important.

## Data Summary

Illumina sequences have been deposited in the European Nucleotide Archive under project PRJEB12513.

**Impact Statement**

Blood stream invasion with the bacterium *Escherichia coli* is increasing across the UK and carries a poor prognosis. About half of these infections are acquired in healthcare settings, with the rest arising from the community. The microbial and host factors that determine the acquisition and outcome of these infections are not known. To uncover some of these factors, we have undertaken whole-genome sequencing of bloodstream isolates of *E. coli* from patients and linked these to their clinical data. We identified the major genetic families of *E. coli* causing bloodstream infection in Scotland. Genetic analysis showed that genes conferring an ability to microbes to survive at low growth rates in the presence of antibiotics were important in hospital-associated infections (HAIs). Genes encoding a mechanism to compete with other microbes were important in community-associated infections (CAIs). Outcome was worse in HAI and was associated with urinary catheter use. Genes encoding resistance to antibiotics used extensively in hospitals clustered together in strains from HAIs, suggesting co-inheritance of multiple resistance genes in these strains. These results show how antibiotic use is driving the evolution of strains causing bloodstream infection in hospitals; factors responsible for CAI are less clear.

## Introduction

The Gram-negative bacterium *Escherichia coli* is a common gut commensal in man, as well as an important cause of a range of intestinal and extra-intestinal infections [1, 2]. It is the leading cause of bloodstream infection across Europe and the USA [3, 4], with a high attributable mortality of between 10–30 % that rises with age [5–7]. Rates of *E. coli* bacteraemia have increased dramatically over the last 10–20 years, rising 25.6 % between 2012/3 and 2016/7 in England and Wales [8], and 46 % between 1992 and 2006 in Denmark [5], with similar increases within the USA [3]. Across Europe, isolates of *E. coli* from blood collected by the European Antimicrobial Resistance Surveillance System increased by 60 % from 2002 to 2008 [4]. About half these bacteraemias are hospital or healthcare associated, with reported percentages of just over 50 % in Scotland in 2016 [9, 10] and in England in 2012/3 [11]. Thus, community-associated infections (CAIs) are also a significant cause of bacteraemias.

The mechanisms underlying these increases in incidence worldwide remain obscure. Data from Europe show that the increase has been more marked in antibiotic-resistant organisms, many of which are multidrug resistant [4]. A more focussed study in England also found an increasing incidence of antibiotic-resistant strains [12]. Because of the rise in incidence of *E. coli* bacteraemias, enhanced surveillance programs have been instituted in both England and Scotland. Preliminary data from the surveillance in England has shown about half of all patients had previous healthcare exposure in the month prior to the bacteraemia [11]. The source of bacteria was estimated to be the urogenital tract in about 50 % of cases. The Scottish data also showed a urinary source in about half of the bacteraemic cases [9].

A number of studies have analysed the genetic make-up of *E. coli* lineages isolated from the urinary tract and/or bacteraemias [2, 13–17]. These have characterized the dominant multilocus sequence types (MLSTs) associated with these infections: sequence types (STs) ST73, ST131, ST95 and ST69 are the most prevalent in both the UK and the USA. ST131 has come to prominence in the last few decades as a widely distributed strain that typically has a multidrug-resistant phenotype [18, 19]. A recent study estimated that ST131 underwent rapid expansion in about 1995 [20]. However, this expansion stabilized quite rapidly, with a new equilibrium established with other *E. coli* STs. Antibiotic resistance is not the only factor involved in the success of *E. coli* strains, since ST73 is the most abundant phenotype found in some studies and is typically antibiotic susceptible. Although whole-genome sequencing has been performed on large collections of clinical isolates, these have lacked detailed clinical data on the source patients and, thus, the clinical factors involved in selection of different bacterial genes that might be associated with the observed increase in rates of bacteraemia and their origin remain unknown.

The ability to link full clinical data with whole-genome sequences of causative microbes greatly increases the ability to identify key pathogen factors involved in acquisition and subsequent development of disease. Here, we have taken advantage of the rich clinical data provided by enhanced surveillance of *E. coli* bacteraemias within Scotland to link this with whole-genome sequencing of the causative strains. Our aim was to explore potential factors associated with the microbe and the host that lead to bacteraemia, and in particular to examine the determinants of CAI versus hospital-associated infection (HAI). A total of 162 bacteraemic *E. coli* isolates from 2013 and 2015 were fully sequenced and analysed in tandem with the clinical data available on each source patient that was recorded as part of an enhanced surveillance program. The results presented here allowed us to examine in depth the population structure of *E. coli* strains causing bacteraemia, and to relate this to the clinical risk factors, antibiotic sensitivities and course of infection. We identified genes important in HAIs versus CAIs by genome-wide association study, which revealed that acquisition of antibiotic tolerance is an important factor in bacterial fitness in healthcare environments. We observed differing virulence determinants between different strains, and evidence of co-selection of multiple antibiotic-resistance genes (ABRs).

## Methods

### Collection of *E. coli* isolates and whole-genome sequencing

A total of 162 blood culture *E. coli* isolates were selected retrospectively from the microbiology laboratories of the Queen Elizabeth University Hospital, Glasgow, Scotland, UK, and Dumfries Royal Infirmary, Dumfries, Scotland, UK. For the Queen Elizabeth Hospital, all non-duplicate isolates in the last quarter of 2013 (97) and from a 6 week period in the last quarter of 2015 (41) were taken. From Dumfries, all non-duplicate isolates from the last quarter of 2013 were collected (24). The total numbers of *E. coli* bacteraemic isolates during these periods were 428 in 2013 and 576 in 2015 from Glasgow, and 95 in Dumfries for 2013. Genomic DNA was extracted at each site using QIAmp (Qiagen) and index-tagged paired-end Illumina sequencing libraries were prepared. Sequencing was performed at the Welcome Trust Sanger Centre (Hinxton, UK) using the 96-plex Illumina HiSeq 2500 platform to generate 2×200 bp paired-end reads. These raw Illumina sequences have been deposited in the European Nucleotide Archive under project PRJEB12513.

### Clinical data for linkage analysis

The clinical data for the Scottish isolates had been collected from blood specimens with patient outcome (e.g. died in hospital or discharged) along with date of death/discharge, catheter association during hospital admission, age, sex, infection type (e.g. urosepsis, bone, skin, respiratory tract or gastrointestinal) and mode of acquisition whether it was a HAI or a CAI. Bacteria isolated from samples taken greater than 48 h after admission to hospital were classified as HAI and those within 48 h of admission to hospital were classed as CAI. Bacterial minimum inhibitory concentration (MIC) and antibiotic-sensitivity data for each isolate were also collected. Multilocus sequence typing analysis of all the isolates was carried out with SRST2 [21], using maximum number of mismatches per read for MLST allele calling as 10.

### Phylogenetic analysis and variant detection

The raw Illumina reads were mapped to the *E. coli* reference genome EC958 (strain ST131, accession no. NZ_HG941718.1) using SAMtools mpileup [22] and were called for single nucleotide polymorphisms (SNPs) through VarScan [23] (read depth ≥2×, variant allele frequency ≥0.08 and *P* value ≥0.005). Mobile genetic elements were masked and recombination filtration was performed using Gubbins [24]. Maximum likelihood (ML) trees were inferred using RAxML [25] with the generalized time-reversible (GTR) model and a gamma distribution to model site-specific rate variation. One hundred bootstraps were conducted for the support of the SNP-based ML phylogenetic tree.

### Pangenome analysis

*De novo* assembly of the Illumina reads and the annotation of the coding sequence regions were conducted using the Sanger pipeline [26]. Core and accessory genes were identified using Roary [27] with blastp identity ≥95 % and percentage of isolates to be in the core genome taken as 99 %. The accessory gene (>5  and <99 % isolates) accumulation curves were generated separately for each of the dominant STs using R. Non-metric multidimensional scaling (NMDS) was performed using the vegan package in R (http://cran.r-project.org/web/packages/vegan/index.html). The variation of core gene and accessory gene content was performed using pairwise distances. For each pair of isolates, we calculated their cophenetic distance from the core gene phylogenetic tree and the Jaccard distance based on presence or absence of accessory genes. These two distances were plotted against each other to compare the diversity of core and accessory genes in Fig. 2(c). Plasmid incompatibility types and subtypes were identified using PlasmidFinder [28].

### Pathway analysis

Genes were extracted from all the isolates using an in-house python script and linked to KEGG (Kyoto Encyclopedia of Genes and Genomes) orthologue or KO groups using david [29]. The fold enrichment for each pathway was evaluated with reference to *E. coli* K-12 genome.

### Virulence genes

Virulence genes were identified using the VirulenceFinder database [30] of SRST2 with percentage of coverage cut-off for calling a gene ≥90, keeping default values for all other parameters. We found 437 virulence genes in our 162 isolates. A Kruskal–Wallis non-parametric test (with Bonferroni correction adjusted *P* values for multiple testing) was performed in R using the stats package.

### Survival analysis

Survival was analysed by the Kaplan-Meier method and comparisons between subgroups were examined by the log rank test using the survival package in R. Cox proportional hazards analysis was carried out to identify the predictors for 6 months mortality that were independent (univariate analysis) or dependent of age and gender (multivariate analysis).

### Genome-wide association study

Pangenome-wide association study was carried out on pangenome genes extracted from all the isolates. Using Scoary [31], the significant genes were identified that were different between the HAI versus CAI groups of isolates. A Kruskal–Wallis significance test with Bonferroni correction was also conducted to reconfirm the significance. The Manhattan plot has genes along the *x*-axis in no particular order and the *y*-axis gives the negative logarithm with base 10 of the significant *P* values.

### ABRs and network analysis

ABRs were detected using ariba [32] using the card database. Altogether 52 ABRs were identified (see the Supplementary Material available in the online version of this article). The correlation networks of ABRs gene presence/absence matrix and clinical susceptibility (R/S) matrices were constructed using Pearson’s correlation and the Hmisc v4.0–2 package in R. The cut-off edge weight value was set at a correlation of >0.5 for the network plot.

## Results

### Study design

We selected isolates of *E. coli* from blood cultures taken from patients within the Greater Glasgow and Clyde Health Board over a 2 month period in 2013 and in 2015. We also collected similar isolates from patients within Dumfries and Galloway in 2013. After removal of potential duplicates, samples that failed to grow and those that failed to provide full genomic sequences, we were left with a total of 162 isolates that were fully analysed as below.

### Population structure of isolates

**Fig. 1.**
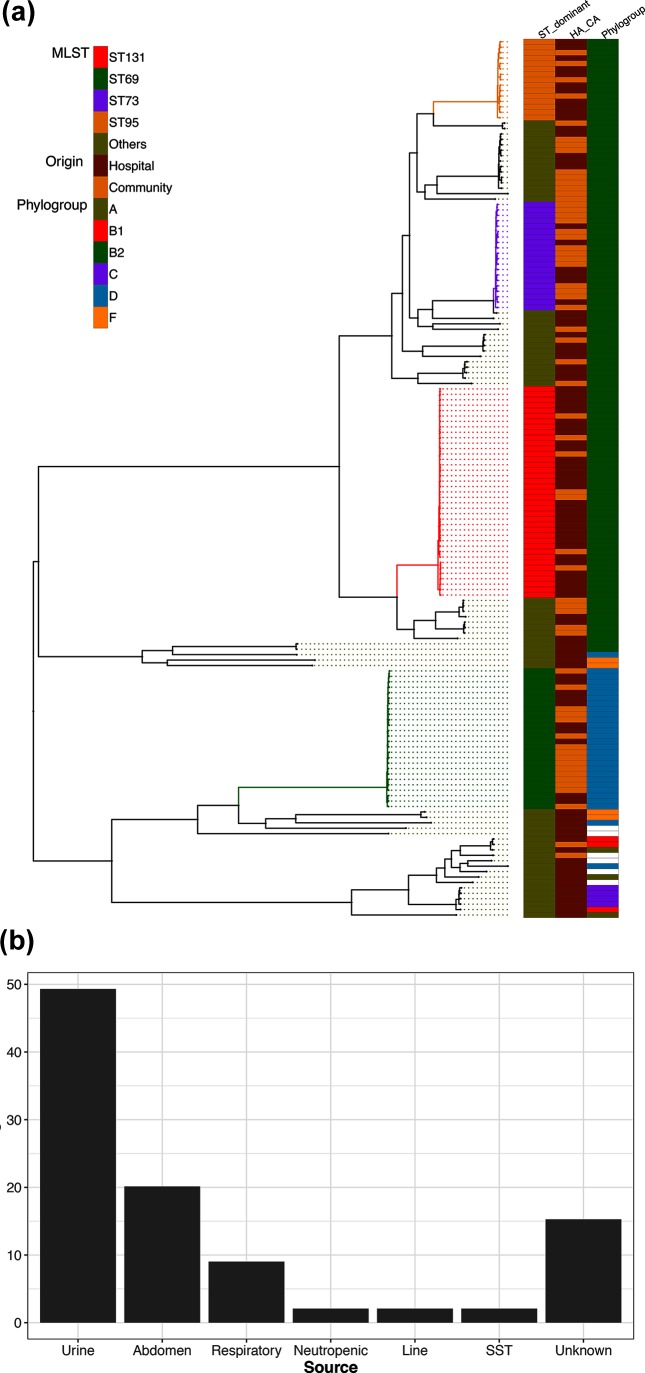
(a) ML SNP core gene phylogeny of the *E. coli* isolates. The STs were identified using the MLST scheme. STs with fewer than 10 isolates are clustered in the category ‘others’. The key indicates phylogroups and whether the isolates were community associated (CA) or hospital associated (HA). (b) Source of bacteraemia as percentages of total isolates. Line, indwelling vascular device; SST, skin and soft tissue.

*De novo* genome assembly produced sequences ranging in length from 4.6 to 5.1 Mbp genomes. A total of 20 855 genes were identified from these assemblies, of which 13 % (2804 genes) were conserved in more than 99 % of genomes. These core genes were used to generate a 2.68 Mbp base pair alignment, which was used to construct a phylogenetic clustering analysis (Fig. 1a). The MLST scheme identified four dominant STs found in more than 10 isolates within our 162 isolates: ST131 (39, 24 %), ST69 (26, 16 %), ST73 (20, 12 %) and ST95 (15, 9 %). Other strains accounted for 38 % of the isolates. The ML phylogenetic analysis, with 100 % bootstrap support, corresponded very well with the MLST identified strain types. The tree was rooted on the longest branch, separating the ST69 strains (phylogroup D) from the other strains ST131, ST73 and ST95, which belong to the phylogroup B2. The source of the bacteraemic isolates was identified by case note review for 144 of the isolates (Fig. 1b). A urinary origin was the commonest source (49 %), followed by abdominal (20 %) and respiratory (9 %) origins.

Sub-analysis of the ST131 isolates into the clades described by Price *et al.* [33] and Petty *et al*. [34] showed that the majority of the ST131 isolates were in clade C (29/39; 74 %), characterized by being of serotype O25H4 and possessing the *fimh30* allele (see Fig. S1). Further subdivision of the C clade was made using clade-specific SNPs as described by Ben Zakour *et al.* [35]. Five isolates were sensitive to ciprofloxacin (C clade). Of the ciprofloxacin-resistant strains, 5 were in clade C1 and 19 in clade C2. Possession of the extended spectrum β-lactamase CTMX-15 has been described as a marker of clade C2. We found 10/19 clade 2 isolates had this gene; in addition, 1 had the gene for CTXM-9. C1 isolates lacked CTXM-15, but one had the CTXM-9 gene. CTXM-15 has, thus, been acquired/lost repeatedly within the C2 clade as noted also by Kallonen *et al.* [20]. One clade A isolate had also acquired the CTXM-15 gene.

### Accessory gene analysis

**Fig. 2.**
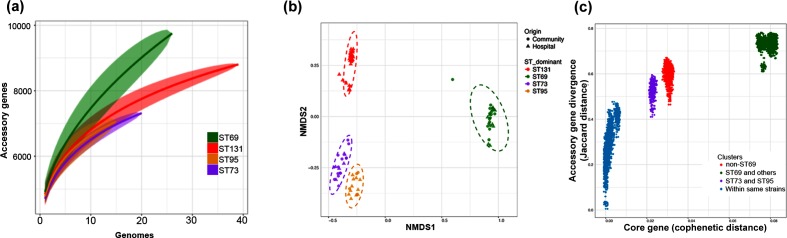
(a) Accessory gene accumulation curves for the dominant STs with respect to the number of genomes compared. At each value *x* of the number of genomes compared, a random sample *n* of genomes was compared in *n*!/(*n−x*)!*x*! ways. The solid line shows the median value at each value of *x*; the shaded area contains the interquartile values. (b) NMDS plots of the different dominant STs based on their shared accessory gene content. Community or hospital origin are as indicated. Dotted lines are 95 % confidence limits of values around the centroid for each ST. (c) Comparison of core and accessory genomes of different STs. Each circle is the core genome similarity as cophenetic distance plotted against accessory gene similarity as Jaccard distance for each possible paired comparison within the different groups as indicated in the key.

We hypothesized that the accessory gene content of the isolates would contain genes important in establishing blood stream infection that were common across all the ST types. Within the 162 isolates, 69 % (*n*=14 328) of the pangenome was identified within the dominant strain types ST131, ST73, ST95 and ST69. We identified 5757 accessory genes that are present in (≥5 and <99 %) our 162 genomes. Most of the accessory genes were rare, and only 22 % have been identified functionally. To measure the size of the accessory genome, we determined the numbers of new genes identified with increasing numbers of genomes sampled for each of the dominant ST types (Fig. 2a). This showed that for all STs, there was a steady rise in the number of new genes identified with increasing sampling. As noted before for *E. coli*, this did not reach a plateau for any of the STs. The accumulation curves fitted a power law as given by Heaps’ law, as described by Tettelin *et al.* [36]. The derived constant α was less than 1 for all ST, indicating an open pangenome. However, as Fig. 2(a) shows, the accumulation curve for ST69 rises more rapidly than the other STs. This is reflected in the derived α constants, which is lowest for the ST69 isolates. Comparing ST69 to the largest group, ST131, the difference in the distributions was significant (two sample Kolgomov Smirnoff test, *P*=0.0071). Overall measures of accessory gene diversity showed all STs were highly diverse, although ST131 and ST69 were marginally more diverse than the other two STs (see Fig. S2).

Next, we assessed whether the accessory gene composition reflected the ST or grouped the isolates in another fashion. Similarity between the accessory gene content of each isolate was analysed using NMDS based on the Jaccard distance of accessory genes between each isolate (Fig. 2b). This showed that isolates grouped according to ST rather than any other parameter. However, there were significant differences in which STs were associated with HAI or CAIs (multinomial goodness of fit test, *P*<0.05, exact binomial probability). ST131 was highly associated with HAI (82 % of isolates, exact binomial compared to overall rate of HAI of 62 %, *P*<0.05) and ST69 significantly associated with CAI (62 % of isolates, exact binomial compared to overall rate of CAI of 38 %, *P*<0.05)

The variation in the accessory gene pool was further assessed by comparing the core genome divergence and accessory gene content in pairwise comparisons (Fig. 2c). Comparison of isolates with other members of the same ST showed little difference in their accessory gene content, clustering towards the origin of the plot (blue dots). Pairwise comparison between all isolates of different ST groups other than ST69 are shown in red, and cluster quite separately from the intra-ST pairwise comparisons. This discontinuity reflects a distinct clonal structure to the population, since higher rates of recombination between STs has been shown to generate a more homogeneous distribution [37]. Comparison of ST69 with all other isolates shows a more extreme clustering in the upper right of the plot (green), highlighting the divergence of ST69 from the other STs, with a significantly different accessory gene content that does not appear to be undergoing exchange from the other STs. This does not reflect any difference between the recombination rates in ST69 compared to the other STs; recombination rates between the different STs were very similar (see Fig. S3). As a comparison, we compared core gene divergence and accessory gene content between ST73 and ST95, which are more closely related in core gene content (purple dots). Accessory gene content was more divergent between these groups than for the intra-strain comparison, but not so extreme as between ST69 and the other STs.

A proportion of accessory genes will be present on different plasmids between the isolates. Although from the short reads provided by Illumina sequencing we were unable to reconstruct plasmids, we identified plasmid profiles within the isolates based on the presence of specific replicon genes that places them in different incompatibility (Inc) groups (Fig. S4). Ten plasmid Inc groups were identified across all the strains. The broad range IncQ plasmids were predominantly found in isolates of ST69 (42 % of the isolates, Fig. S4); the differences between the groups were significant by G test of independence (*P*<0.001 with Williams correction), with significant increases in the percentage of IncQ plasmids in ST69 compared to ST131 and ST73 (G test false discovery rate corrected *P* values of 0.002 and 0.01, respectively). Other plasmids without recognized replicon genes may also be present in these strains.

**Fig. 3.**
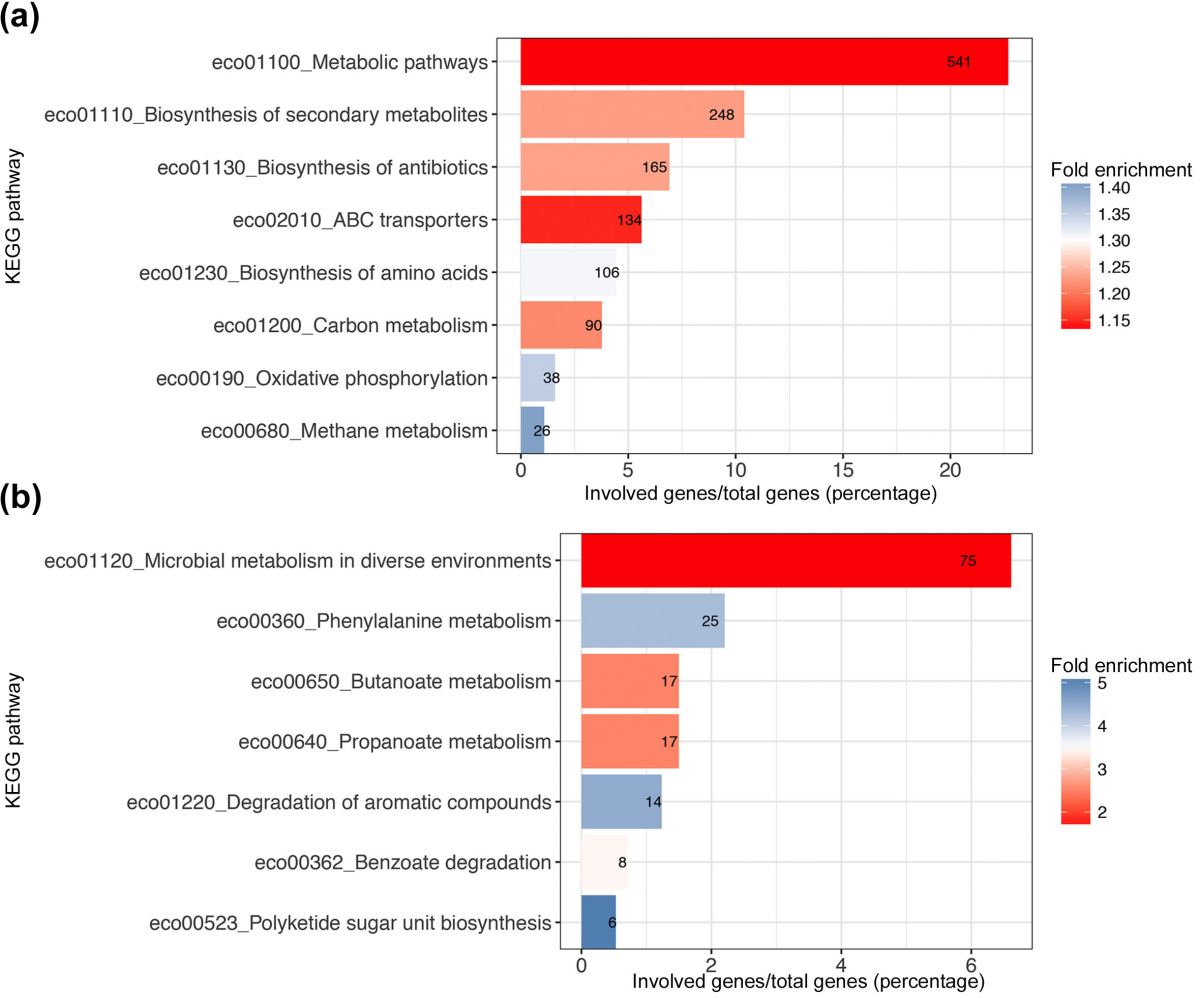
KEGG pathway analysis of (a) core and (b) accessory genes of the different isolates. Comparison was made to the KEGG assignation of genes with the *E. coli* reference strain K12. Bars show the genes significantly associated within each KEGG group as a percentage of the total number of the core or accessory genes with a KEGG pathway assignation. Fold enrichment of the different groups is as indicated in the colour key. Superimposed numbers are the number of genes assigned within the indicated KEGG pathway.

We compared the pathway designations of the core and accessory genomes of the different isolates (Fig. 3). Compared to the *E. coli* reference strain K12, the core genome showed modest but significant enrichment principally of metabolic pathways (Fig. 3a). The accessory genome had over-representation of different pathways (Fig. 3b). These included phenylalanine metabolism, products of which have been implicated in bacterial pathogenesis [38]. We also determined which genes were common in the accessory genome of the different isolates but not at the 99 % threshold of the core genome. A total of 495 genes were identified as shared between 80 % of the commonest isolates ST131, ST69 and ST73. Pathway analysis of these genes did not reveal any significant common pathways.

### Virulence genes

**Fig. 4.**
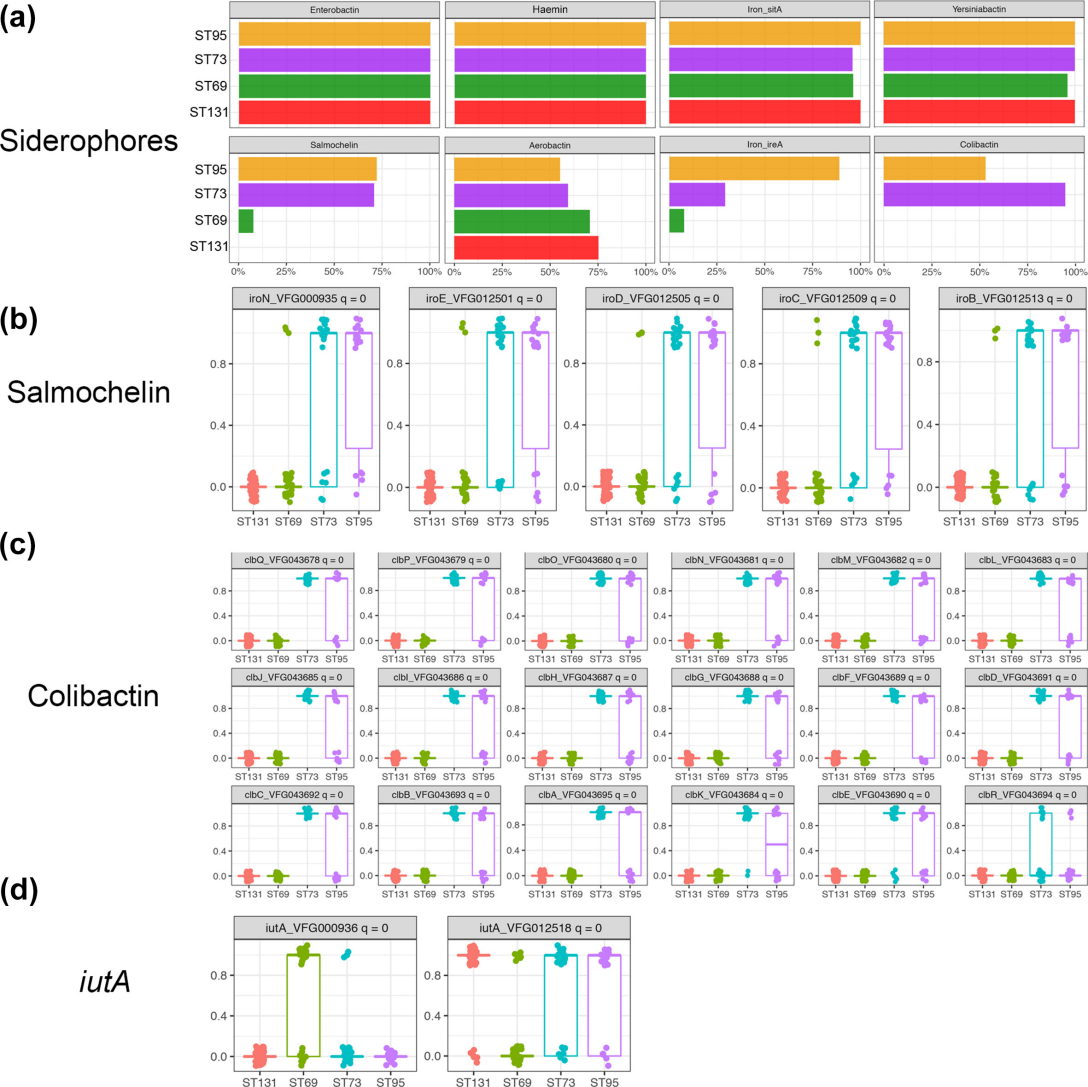
Distribution of siderophores within the major STs. (a) Percentage of each major ST containing the genes for the indicated siderophore systems. (b–d) Individual occurrences of each indicated siderophore gene within the different STs. Genes are scored as present (1) or absent (0), with each circle representing a single isolate; symbols are dodged to aid in viewing. Calculated q values are shown in each graph header; values <10^−6^ are assigned as 0.

We used the Virulence Factor Database [39] to determine the different virulence genes within the different *E. coli* strains. A total of 363 different virulence genes were detected. In keeping with the predominantly urinary origin of about 50 % of the isolates, genes previously identified as important in urinary pathogenic *E. coli* were highly prevalent. Genes encoding siderophores showed considerable variation between the different dominant ST strains (Fig. 4). Genes encoding enterobactin, haemin, yersinobactin and the metal transporter SitA were found in virtually all of the dominant STs. However, genes for salmochelin and colibactin were almost exclusively present in ST95 and ST73 (Fig. 4). Fig. S5 (a–d) shows the distribution of individual siderophore genes between the STs. Genes of the salmochelin operon (*iroBCDEN*) were present together in the salmochelin-positive strains. Likewise, genes of the colibactin operon were also found within ST95 and ST73. Two different alleles of the *iutA* gene that is involved in aerobactin synthesis were distributed differentially between the different STs; the allele VFG012518 has been found in plasmids isolated from avian pathogenic *E. coli* strains, suggesting possible transmission of plasmids or elements of these plasmids between humans and farmed poultry [40].

**Fig. 5.**
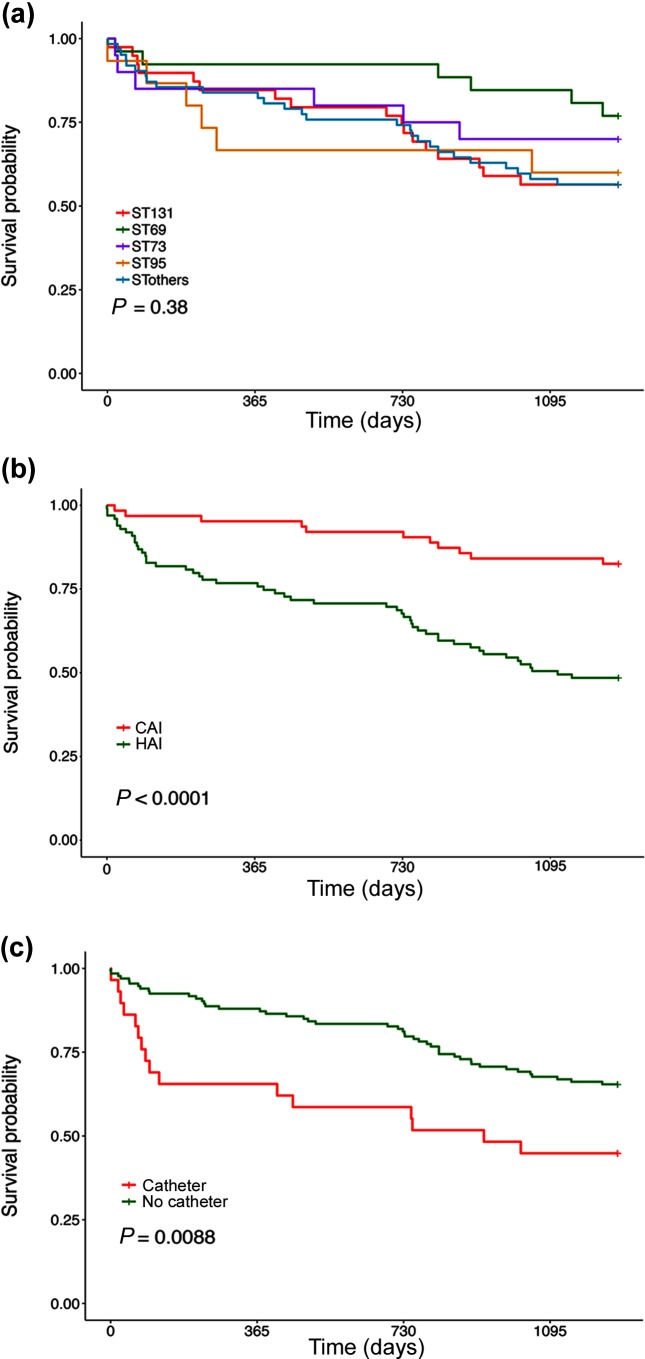
Kaplan–Meir survival curves of the patients within the study stratified according to (a) ST, (b) CAI or HAI and (c) presence or absence of urinary catheter. *P* values show the significance of the differences between the outcomes using the Cox proportional hazards model.

Virulence genes that showed a significant difference between the dominant STs are shown in Figs S5 and S6. The diversity of virulence gene content between the different STs is very clear. Some of these genes were only found within one ST. Numerous adhesins that are known to be important in the pathogenesis of urinary infection were present in these isolates, and some significant differences were detected. Some of the divergence is associated with different alleles of adhesion genes, such as *papG,* which is the terminal component of P type pili that is key in mediating attachment of bacteria to receptors within the kidney and initiating pyelonephritis. This allele (vfg000890) is a class II *papG* that is known to be important in attachment of *E. coli* to cells in the upper urinary tract and in the development of sepsis [41, 42]. However, some of the ST73 isolates were unique in possessing genes encoding components of the S and FIC pili [43], which were not seen in any of the other isolates. There were significant differences in the presence of members of the serine protease autotransporters of Enterobacteriacae (SPATE) family [44]. A previous study of ST131 found the SPATE family gene *espC* in 99 % of ST131 isolates [20]; however, we only found one of our isolates contained this gene, which was an ST131. ST69 strains contained many genes that are part of a second type III secretion system (ETT2) [45] previously described in strains of enterohaemorrhagic and enteroadhesive *E. coli,* which we found intact in a complete operon. Finally, members of strains ST73 and ST131 possessed genes of the haemolysin operon, a toxin important in the pathogenesis of urinary *E. coli* infections [46].

### Outcome and risk factors for death

To begin to dissect the contribution of this diverse genomic repertoire to disease, we compared the outcome of patients infected with the different dominant ST types (Fig. 5a). The Kaplan–Meir survival curves show little difference between the different STs in mortality; ST69 shows a slower rate but multiple comparisons did not reveal statistically significant differences (Cox proportional hazards model with correction for multiple testing). Mortality between HAI and CAI showed a significant difference (Fig. 5b). HAIs showed a worse outcome; the curves separated markedly in the first 90 days after the *E. coli* was isolated and then became parallel. We also tested whether presence of a urinary catheter influenced outcome (Fig. 5c). Early mortality was significantly more marked in patients who had a urinary catheter at some point in their hospital stay. We performed univariate and multivariate analysis of 6 month mortality in this group of bacteraemic patients with a number of clinical factors (Table 1). Age, presence of a urinary catheter and a respiratory source were all significant risk factors for 6 month mortality in a univariate analysis. Presence of a urinary catheter and respiratory source remained significant in a multivariate analysis.

**Table 1.** Univariate and multivariate analysis for predictors of 6 months mortality calculated on 162 isolates *P* values (*P* val) and confidence intervals (CI) are shown. *P* values less than 0.05 are shown in bold.

	Univariate analysis			Multivariate analysis		
Variable	Odds ratio	*P* val	95 % CI	Odds ratio	*P* val	95 % CI
Gender – female (ref: male)	0.91	0.8369	0.37–2.23			
Age>65 (years)(ref: ≤65 (years))	3.66	***P*=0.0384**	1.07–12.48			
Hospital (ref: community)	1.73	0.2627	0.66–4.49	1.62	0.3411	0.60–4.34
Urinary catheter (ref: no catheter)	4.83	***P*=0.0005**	1.97–11.83	6.22	***P*=0.001**	2.09–18.48
Diagnosis (ref: gastrointestinal)						
Urosepsis	1.83	0.4371	0.40–8.33	1.34	0.7121	0.27–6.45
Respiratory	7.03	***P*=0.0243**	1.29–38.43	5.55	***P*<0.05**	0.97–31.499
Others	1.36	0.7210	0.25–7.44	1.91	0.4873	0.31–11.95
Strain types (ref: 0)						
ST131	0.78	0.6531	0.26–2.33			
ST69	0.57	0.4451	0.13–2.44			
ST73	1.30	0.6735	0.38–4.44			
ST95	1.10	0.8982	0.26–4.47			

### Determinants of HAI versus CAI

**Fig. 6.**
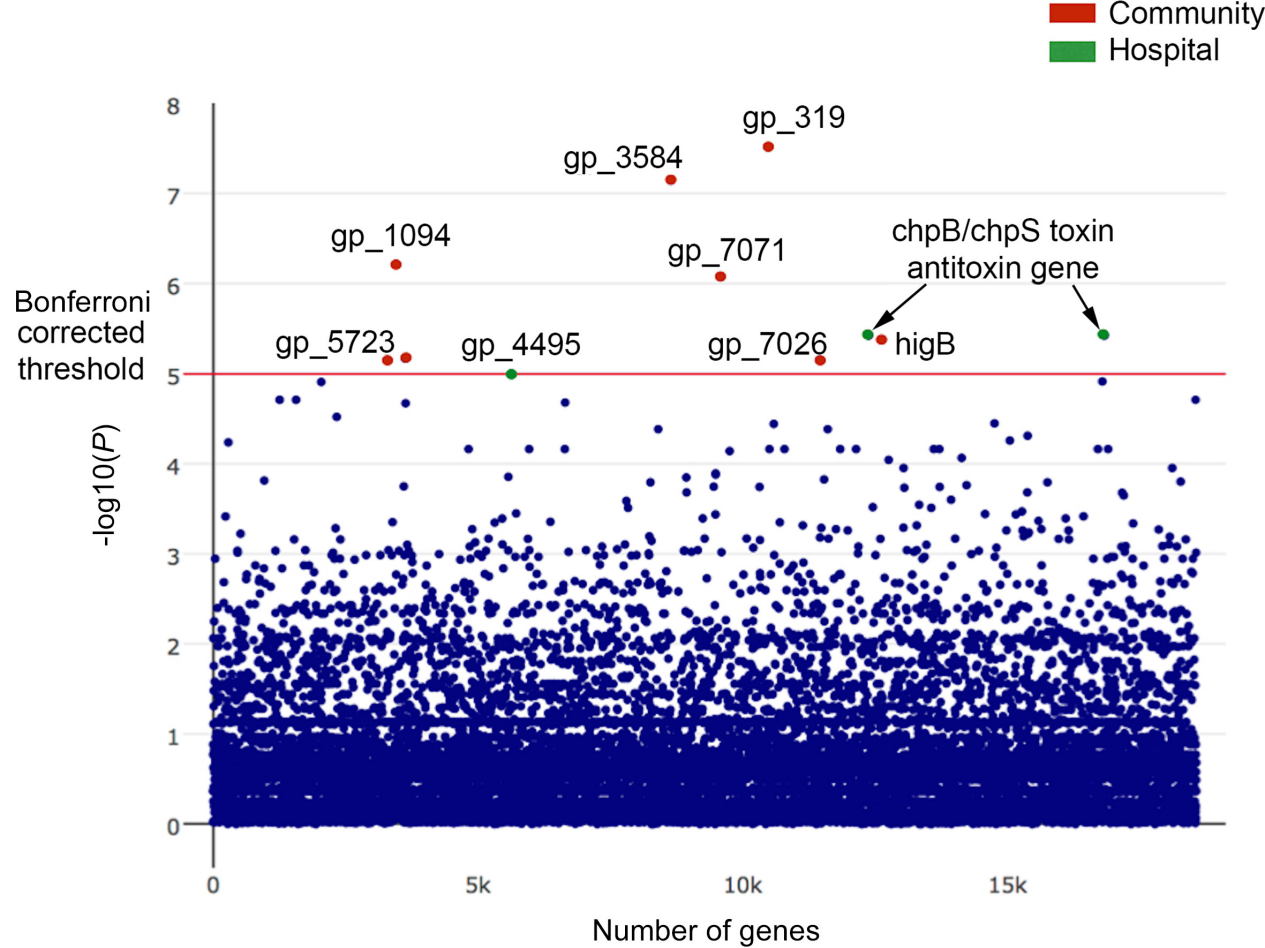
Manhattan plot of association of genes with CAI or HAI detected by a genome-wide association study. Association of genes with phenotype was determined by multiple Kruskal–Wallis tests. Uncorrected *P* values are shown with the Bonferroni corrected *P* value for significance threshold indicated by the red horizontal line. Genes more highly represented in HAI are shown in green and those more highly represented in CAIs are in red. Individual distributions of Bonferroni corrected significant associations are shown in Fig. S7.

Sixty-two per cent of all the bacteraemic isolates were from HAI. We wished to discover the determinants of HAI versus CAI using the whole-genome sequence data. To analyse the determinants within isolates that favour healthcare-associated or community-acquired bacteraemia, we performed a genome-wide association study to identify those genes preferentially present in the two different groups (Figs 6 and S7). We used the software programme Scoary [31], which scores the pangenome of isolates associated with phenotypic characteristics while accounting for population stratification. This correction is important, as spurious associations of genes with phenotypic characteristics may arise because of the strong association of some genes with different STs in *E. coli*.

In the healthcare-associated bacteraemic isolates, we did not find any significant association with genes mediating antibiotic resistance, but there was a significant association with the *chpB/chpS* genes encoding a chromosomal toxin/antitoxin pair. ChpB is a toxin of a type II toxin/antitoxin module that is an endoribonuclease, inhibiting protein translation and inducing bacterial stasis [47, 48]. Such chromosomal toxin/antitoxin modules are increasingly recognized as crucial in the phenomenon of bacterial persistence [49]. These are cells that enter into a dormant non-growing state on exposure to antibiotics or other adverse conditions that allows them to survive until such environmental stress is relieved. Another gene found significantly more frequently in isolates from HAIs is *BglH* (*YieC*), which encodes an outer-membrane carbohydrate-specific porin [50]. In isolates from CAIs, a number of genes associated with a type VI secretion system were identified: an Rhs/Vgr-family protein (gp_319), and a PAAR-repeat-containing protein (gp_3584). Additionally, the gene *higB-1* was more common in the community-acquired isolates; this encodes a putative mRNA interferase as part of a toxin/antitoxin pair [51].

### Resistance to antimicrobials

**Fig. 7.**
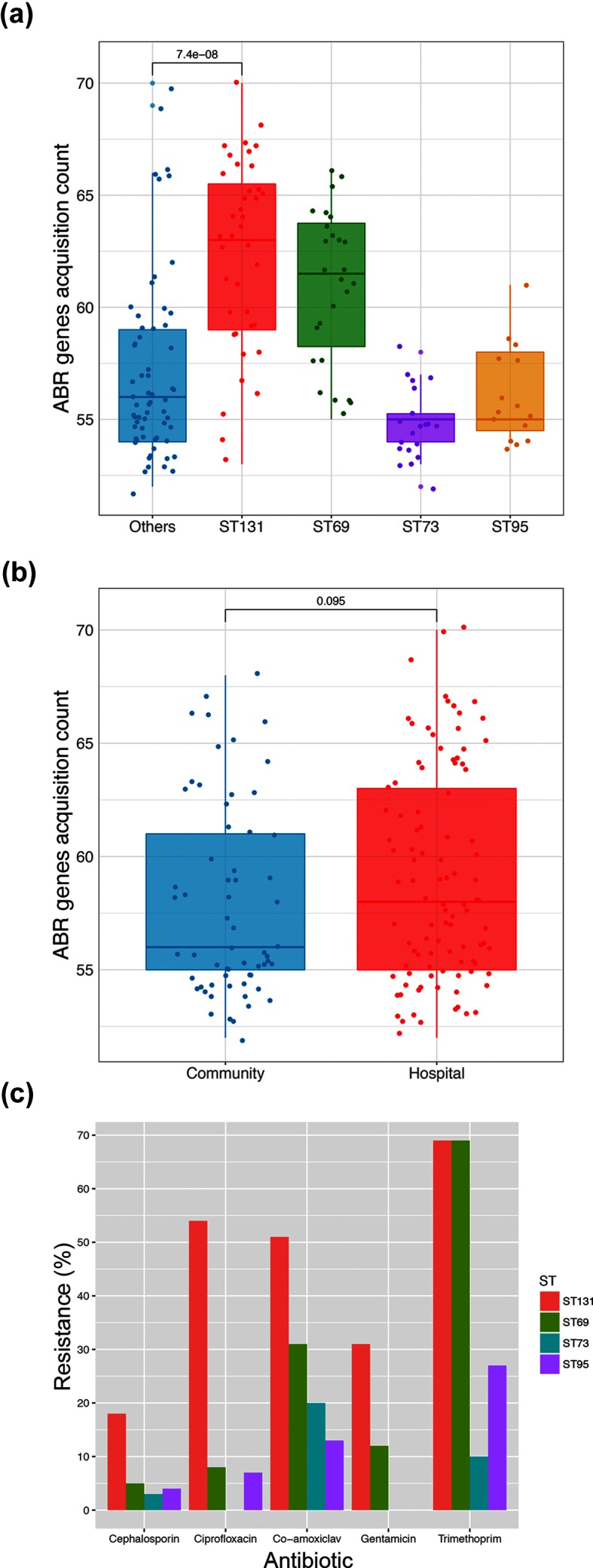
ABRs and phenotypes within the isolates. (a) Distribution of ABRs within the different STs. Each circle is the value of single isolate; boxes show the interquartile range and the horizontal line the median. * indicates a significant difference between ST131 and others, with *P* value as shown. (b) Distribution of ABRs between isolates from community or hospital sources. Symbols as in (a). There was no significant difference between the groups. (c) Percentage of dominant STs having phenotypic resistance to the indicated antibiotics.

In the collection, 98 resistance genes were identified; their distribution is shown in Fig. S8. Mutations conferring ciprofloxacin resistance were also present. There were significantly higher numbers of ABRs in ST131 and ST69 compared to the other STs (Fig. 7a). However, there was no difference between the ABR counts between isolates from HAI and CAI (Fig. 7b). We compared phenotypic resistance to five key antibiotics between the four main STs: 3rd generation cephalosporins, ciprofloxacin, co-amoxiclav, gentamicin and trimethoprim (Fig. 7c). This revealed significant differences between the main STs (overall G test, *P*=1.84×10^−11^); differences between all possible pairs were also significant (G test pairwise nominal test for independence with false discovery rate correction, *P* values all <0.05). ST131 was notable for significant antibiotic resistance in all of these classes, as has been noted elsewhere [19]. In particular, some ST131 strains contained extended spectrum β-lactamases of the CTX-M class, which mediate resistance to 3rd generation cephalosporins. The predominantly community-associated ST69 showed high levels of phenotypic resistance to co-amoxiclav (31 % of isolates) as did ST131 (51 % of isolates). Both ST131 and ST69 showed a high percentage of resistance to trimethoprim (69 % of isolates for both STs).

**Fig. 8.**
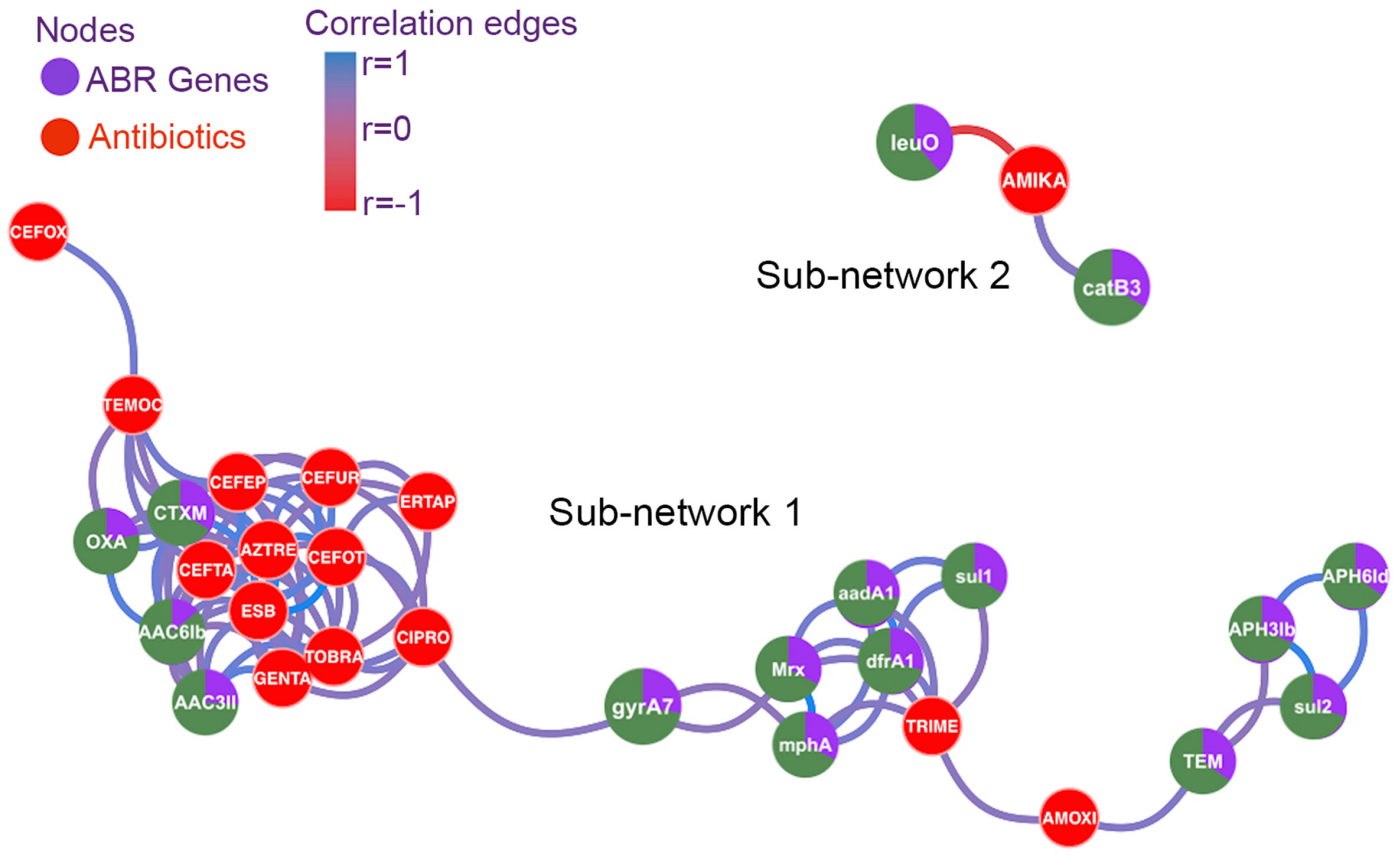
Network analysis of ABRs and phenotypic resistance. The network is based on Pearson correlation coefficients between the different determinants. Only correlations with absolute value >0.5 are shown, colour coded as indicated. Strength of correlation is also coded by proximity of nodes. Genes are shown in green/purple with the green sector indicating the proportion of these genes found in hospital-acquired isolates. Phenotypic resistance nodes are coloured red.

We wished to determine whether there were significant correlations between phenotypic resistance to certain antibiotics and the presence of known ABRs. We hypothesized that increased levels of antibiotic use as found for HAI might result in selection for multiply resistant strains. Moreover, the common organization of ABRs within integrons [52] and within plasmids [53] could result in the co-selection of many different ABRs as a group. We tested this hypothesis by calculating the Pearson correlation coefficient between presence and absence of ABRs within strains and antibiotic susceptibility, coded as sensitive or resistance. The correlation matrices were visualized with a network plot (Fig. 8). Nodes for ABRs are shown in green/purple, and phenotypic resistance in red. Linking edges show the degree of correlation; the higher the degree of correlation, the shorter the edge. Only correlations with a value >0.5 are shown. The plot shows a number of key relationships. Firstly, there is a marked strong correlation between antibiotic resistance to a group of antibiotics whose use is largely confined to hospital use, such as gentamicin, tobramycin, aztreonam, ertapenem and 3rd generation cephalosporins (all clustered closely to the left of the figure). A number of ABRs are associated with these phenotypic resistances. These include extended spectrum β-lactamases of the CTXM class as well as β-lactamases of the OXA class that are associated with resistance to co-amoxiclav [54]. Aminoglycoside-resistance genes are also contained within this group: *aac(3′)II* encodes an aminoglycoside *N*-acetyltransferase that mediates resistance to gentamicin and tobramycin, while *aac(6)Ib* encodes a protein that mediates resistance to gentamicin and other aminoglycosides [55]. On the right of the network is a looser cluster of ABRs and phenotypic resistances. These are centred around amoxicillin and trimethoprim, both antibiotics with extensive community use. Trimethoprim resistance is linked to the *dfrA1* gene, a dihydrofolate reductase allele that is not inhibited by trimethoprim. However, there is close correlation also with the streptomycin/spectinomycin resistance gene *aadA1*, genes within an erythromycin-resistance operon (*mrx* and *mphA*), and *sul1*, a sulphonamide-resistance determinant. This would seem to be an example of co-selection, as these genes are commonly found linked as part of an integron [56]. These alleles were extremely closely linked; of 51 isolates with the *dfrA1* trimethoprim-resistance gene, 47 also contained the *aadA1* gene. Similar considerations apply to the linkage between the β-lactamase of the TEM class that mediates amoxicillin resistance, and *aph3Ib* and *aph6Id*, which mediate kanamycin/streptomycin resistance, and *sul2,* which mediates sulphonamide resistance. *sul2*, *aph3Ib* and *aph6Id* are commonly genetically linked on DNA fragments found in plasmids, integrative elements and chromosomal islands [55].

A separate sub-network links amikacin resistance to the genes encoding transcriptional regulator *leuO* and *catB*3, which mediates chloramphenicol resistance. LeuO has been shown to influence expression of a number of operons involved in drug resistance [57]. The linkage between *catB3* and amikacin resistance is not clear.

## Discussion

We have analysed whole-genome sequences of *E. coli* isolated from blood cultures of patients in Scotland in 2013 and 2015, and linked them to clinical data on the same patients. This has allowed us to explore the relationships between genes within these isolates and the origins and clinical course of these infections, as well as detailed information on the range of strains producing these infections and their sensitivity to antibiotics. The four most prevalent STs found in our study – ST131, ST69, ST73 and ST95 – are similar to those in reports of isolates from elsewhere in the UK, Ireland and the USA [2, 13–17, 20]. About half of our isolates had a urinary origin and this was reflected in the content of virulence genes, which contained many genes known to be involved in the pathogenesis of urinary infection and pyelonephritis. Most of the isolates were from the known virulent phylogroups B2 and D [58].

A recent study of isolates from the UK showed the appearance of ST131 and ST69 from about 2002 [20]. ST131 was the commonest ST in our study and was more prevalent in hospital-acquired bacteraemias, as found in other studies [19]. ST73 was only the third most prevalent ST (12 %) of our isolates, compared to a recent study of bacteraemic isolates in England from 2001 to 2011 in which it was the most common isolate [20]. ST69 was the second commonest ST in our isolates and shows some significant differences from the other STs causing bacteraemias. ST69 was more common in CAI and diverged the most from the other dominant STs in its core gene content as well as its accessory genome (Fig. 2). The marked divergence of ST69 accessory genome from the accessory genomes of the other dominant STs suggests very little recombination is occurring with these other STs. We also found a higher content of plasmids with IncQ replicons within ST69 compared to the other STs. Taken together, these differences highlight that ST69 is significantly distinct from the other STs and would appear to occupy a rather distinct ecological niche compared to the other STs.

Virulence determinants were widely distributed within the isolates. However, no common pattern of such determinants was found. Rather, there are multiple different combinations of such factors within bacteraemic isolates that segregate between STs rather than any other factor. Although some determinants were found in several of the dominant STs, some were only present in one ST. This suggests that these factors were either acquired subsequent to the divergence of the different STs or lost in those that no longer possess the factor. It is difficult to determine which of these alternatives have given rise to the observed lineages. Establishing the ancestral history of *E. coli* is problematic because of the high degree of recombination and horizontal gene transfer [59]. Loss of gene function can allow a pathogen to be better adapted to a new niche [60]. ST69 was unusual in possessing an intact second type III secretion system (ETT2) [45] previously described in strains of enterohaemorrhagic and enteroadhesive *E. coli,* which we found intact in a complete operon. What contribution this might make to the pathogenesis of bacteraemia produced by this ST is not clear. That most other *E. coli* strains contain degenerate versions of the ETT2 [45] suggests it is ancestral in ST69. Genes that were present in isolates of one or two STs were more likely to have been acquired during evolution of the various strains.

Analysis of the accessory gene content of the different isolates gave a similar result as seen with analysis of the virulence genes. We had hypothesized that there might be a ‘common’ set of accessory genes present in our isolates that would be important in producing bacteraemia. This was not the case; no particular functional class of genes were found in the accessory genome. The accessory gene pool of *E. coli* is open, essentially unbounded. This strongly suggests that different STs must exist in different ecological niches, as they do not share significant accessory genome content. The origin of these pathogenic strains is most likely from strains carried within the human gut that then colonize the urinary tract and from there invade the bloodstream. The maintenance of distinct accessory genomes between the different STs could be explained by a number of different mechanisms. Colonization of the human gut from another source may be in evolutionary terms a too recent event for significant gene transfer between the different STs to have occurred. Alternatively, these pathogenic strains may be only transiently present in the gut microbiota and are replaced from time to time from another source. Studies of faecal carriage of extended spectrum β-lactamase-producing *E. coli* in patients who had a urinary infection with these pathogens show that they are cleared from the gut over a period of a few years [61]. Whether the same holds for non-extended spectrum β-lactamase producing STs is not clear. Hospitals might provide an environmental source for reinfection of patients, but the origin of community-acquired *E. coli* infections is not clear.

We used the whole genome sequences of the bacteraemic isolates to identify genetic determinants that are associated with CAI versus HAI. Chromosomal genes encoding the toxin/antitoxin pair *chpB*/*chpS* were strongly associated with HAIs. Toxin/antitoxin pairs were originally described in plasmids, where they act as ‘addiction mechanisms’ for plasmid maintenance. Continued expression of the labile antitoxin is required to ensure the more stable toxin is neutralized. Thus, plasmid loss results in unfettered expression of the more stable toxin and death of the cell, ensuring a strong selection pressure for plasmid maintenance. A number of chromosomal toxin/antitoxin pairs have also now been described. Such toxin/antitoxins are important in mediating the phenomenon of bacterial persistence, survival in the face of adverse environmental conditions such as antibiotic exposure [47, 62]. Stress results in protease destruction of the antitoxin molecule, resulting in unchecked toxin activity, which produces growth inhibition. When the stress is relieved, the bacteria recover and can resume their normal growth. This is not antibiotic resistance, but importantly has been found to be closely associated with the onset of true antibiotic resistance in laboratory experiments [63]. We speculate that the association of genes conferring antibiotic tolerance in HAI reflects the general burden of antibiotic use in this setting. Selection of these genes then will facilitate the subsequent generation and selection of ABRs in these isolates. Community-acquired strains showed an increase in genes encoding components of the type 6 secretory systems present in *E. coli.* Studies have shown that this system is a potent mediator of inter-species killing that allows the organism to compete more effectively with other bacteria to establish colonization [64]. This may be important to allow strains such as ST69 that are more frequently the cause of CAIs to establish colonization within the gut and/or urinary tract. Together with the data showing the genetic divergence of ST69 from the other STs that cause bacteraemias, this supports the view that ST69 occupies a distinct ecological niche and has evolved adaptations favouring its survival within the community and the ability to cause bacteraemia. We hypothesize that type VI secretion by ST69 may be an important factor allowing this successful adaptation and could potentially be exploited therapeutically.

Antibiotic resistance was highly prevalent in the isolates examined here. Our network analysis of resistance genes and phenotypic resistance of isolates revealed two main clusters. There was a strongly correlated cluster of genes conferring phenotypic resistance to typical ‘hospital’ antibiotics, such as third-generation cephalosporins, aminoglycosides and β-lactamase-resistant penicillins. This is strong evidence that within this setting, use of these antibiotics is generating cross-resistance to other unrelated antibiotics, most likely through selection of plasmids containing multiple antibiotic-resistance determinants. This phenomenon also is seen with the predominantly ‘community’ utilized antibiotics trimethoprim and amoxicillin. However, in these cases, the co-selected genes mediate resistance to aminoglycosides that are not currently used to any degree in Scotland (e.g. streptomycin). However, if, say, an integron mediating trimethoprim resistance were to acquire a resistance gene to a more commonly used antibiotic, then this could be strongly selected by trimethoprim use.

The CTXM-15 extended spectrum β-lactamase was found almost exclusively in ST131 isolates of clade C2, but was present in one isolate ST131 clade A and one isolate of ST405. This gene is widespread in isolates of ST131 and is of high clinical significance given that it confers resistance to the widely used 3rd generation cephalosporins cefotaxime and ceftazidime. CTX-M15 has also been reported in ST405 and other isolates [65]. The CTXM group of genes originated from chromosomal β-lactamases present in species of the microbial genus *Kluyvera*. The spread within ST131 occurred during the 1990s and it is now present worldwide [66], but its presence of this gene in ST405 and other STs suggests that it has the potential to become even more widespread.

Outcome of the patients studied here after the isolation of the infecting *E. coli* showed no significant dependence on ST. Patients with HAIs fared worse than those with infections acquired from the community; the excess mortality was in the first 90 days after infection suggesting a causal link. Patients who had a catheter also fared worse in the initial 90 days. However, it proved difficult to establish from patients’ notes exactly when catheters were placed. Since more severely ill patients may be catheterized by preference, the causal link with catheterization will require further study. In multivariate analysis, apart from catheterization, a respiratory source of infection was also a risk factor for death within 6 months. Previous studies have identified non-urinary source as a risk factor for death [67], and a comprehensive study in France showed a number of host factors and portal of entry were more important than bacterial determinants as predictors of death [68]. The association with a respiratory source has not previously been specifically identified.

In conclusion, this study has revealed the complex genetic patterns present in *E. coli* isolates from bacteraemic patients in the West of Scotland in 2013–5. We identify ST69 as an important contributor to the burden of disease, showing that it is predominantly a CAI with a distinct genetic make-up. Using a genome-wide association study, we have identified genes mediating antibiotic tolerance as important in HAI and elements of a type VI secretion system in CAI. The close correlation of resistance to multiple antibiotics that are largely used in hospitals, show how antibiotic use is driving selection of such resistance determinants, which subsequently can spread to community isolates. It is unlikely given the diverse mechanisms by which these strains of *E. coli* can cause disease that a single intervention will reduce the incidence of such infections. However, this study has identified a number of factors that could be targeted to reduce the burden of this disease.

## Data bibliography

1. Scottish Healthcare Associated Infection Prevention Institute (SHAIPI). European Nucleotide Archive, PRJEB12513 (2017).

## Supplementary Data

1595 Supplementary File 1
