# Supplementary material for: Genetic analysis of invasive Escherichia coli in Scotland reveals determinants of healthcare-associated versus community-acquired infections
Source: Microb Genom. 2018 Jun 22;4(6):e000190. doi: 10.1099/mgen.0.000190 (PMC6096937; doi:10.1099/mgen.0.000190)
Supplement: Supplementary File 1 [file mgen-4-190-s001.pdf]

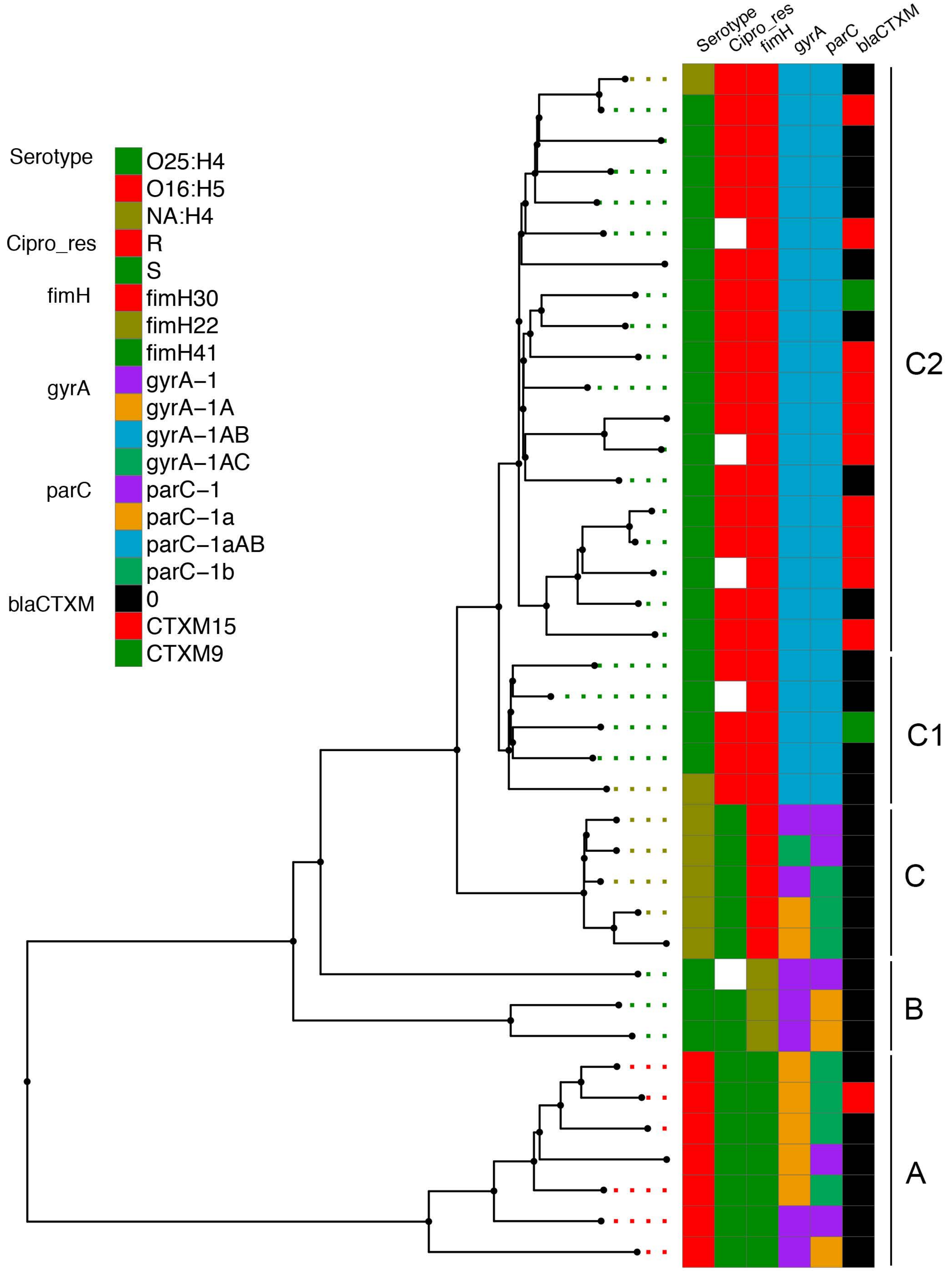

Figure S1. Maximum-likelihood phylogenetic tree based on SNPs within core genome of the ST131 isolates. Isolates are grouped into the A, B C, C1 and C2 clades as described in the text based on their serotype, resistance to Ciprofloxacin (Cipro_res) fimH allele, gyrA and parC genotype and presence of a CTXM extended spectrum beta lactamase, as indicated.

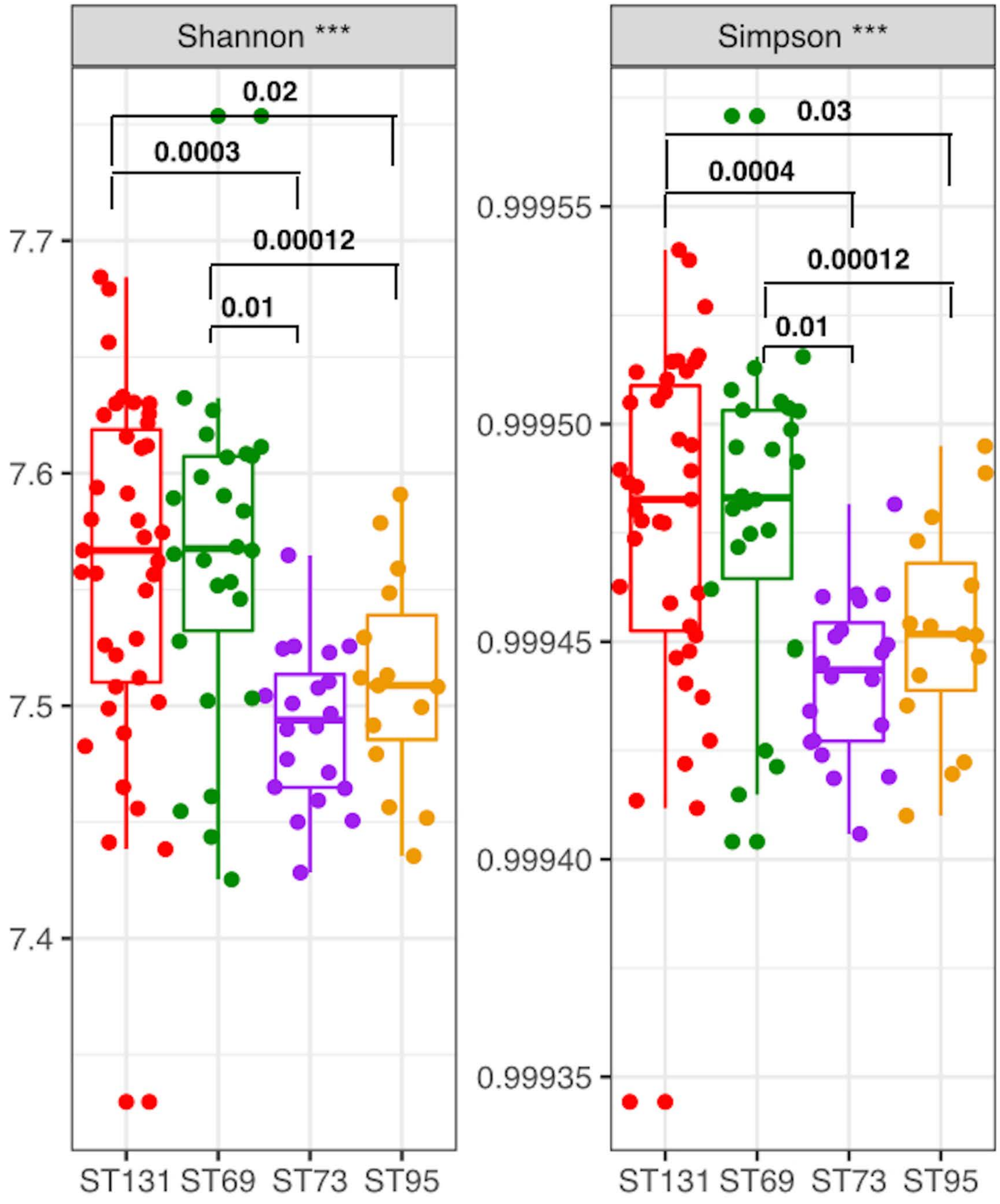

Figure S2. Diversity indices of accessory genes as shown for the dominant STs. Each circle is the value from a single isolate; boxes show the interquartile range and the line the median. P values of the pairwise comparisons are as shown.

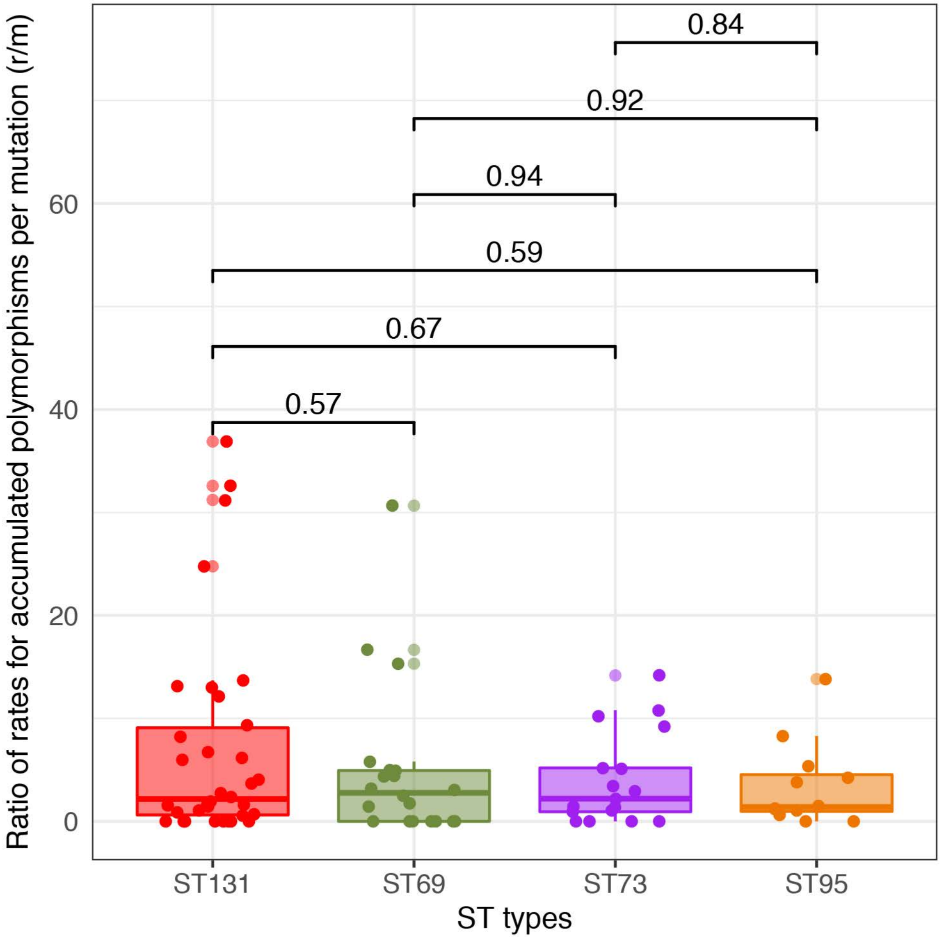

Figure S3. Recombination rates relative to single base mutations (r/m) for the dominant STs. Symbols as in Fig S2.

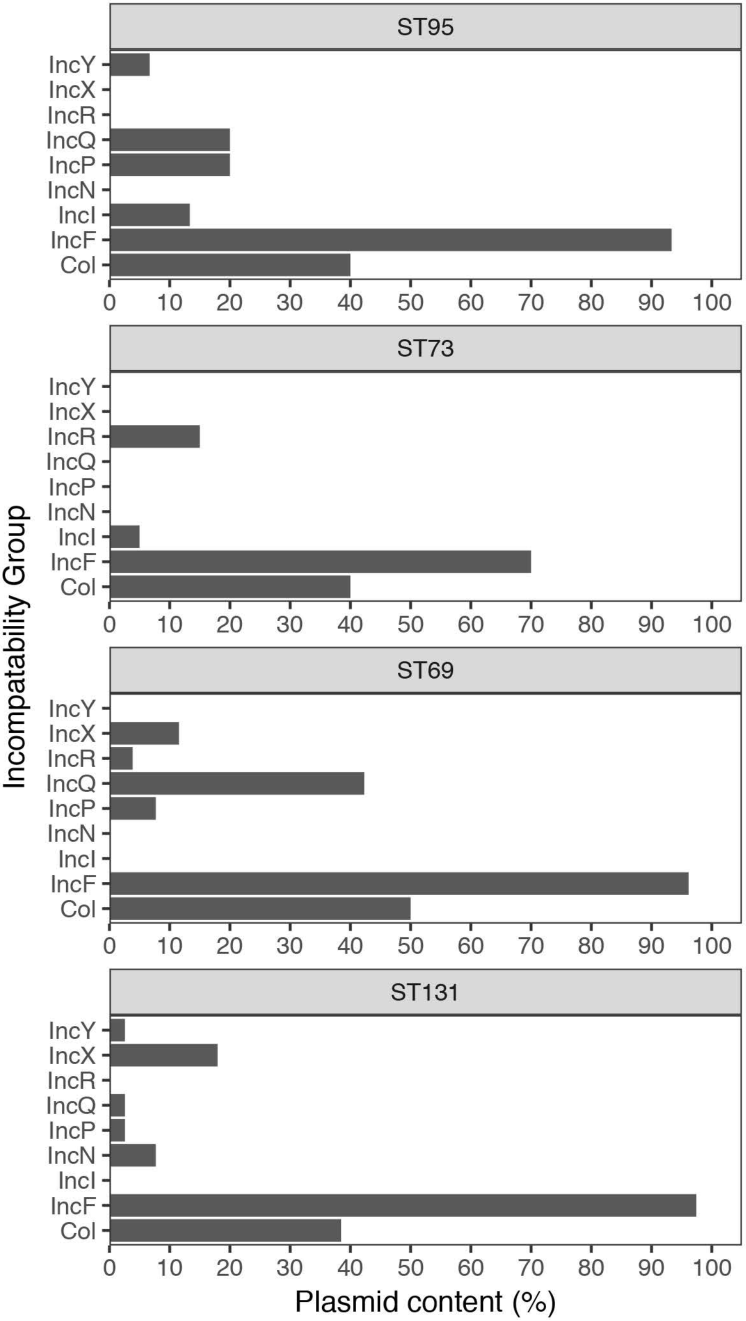

Figure S4. Distribution of different plasmid incompatibility groups between the different dominant STs. X axes show the percentages of each different ST containing the indicated plasmid incompatability group.

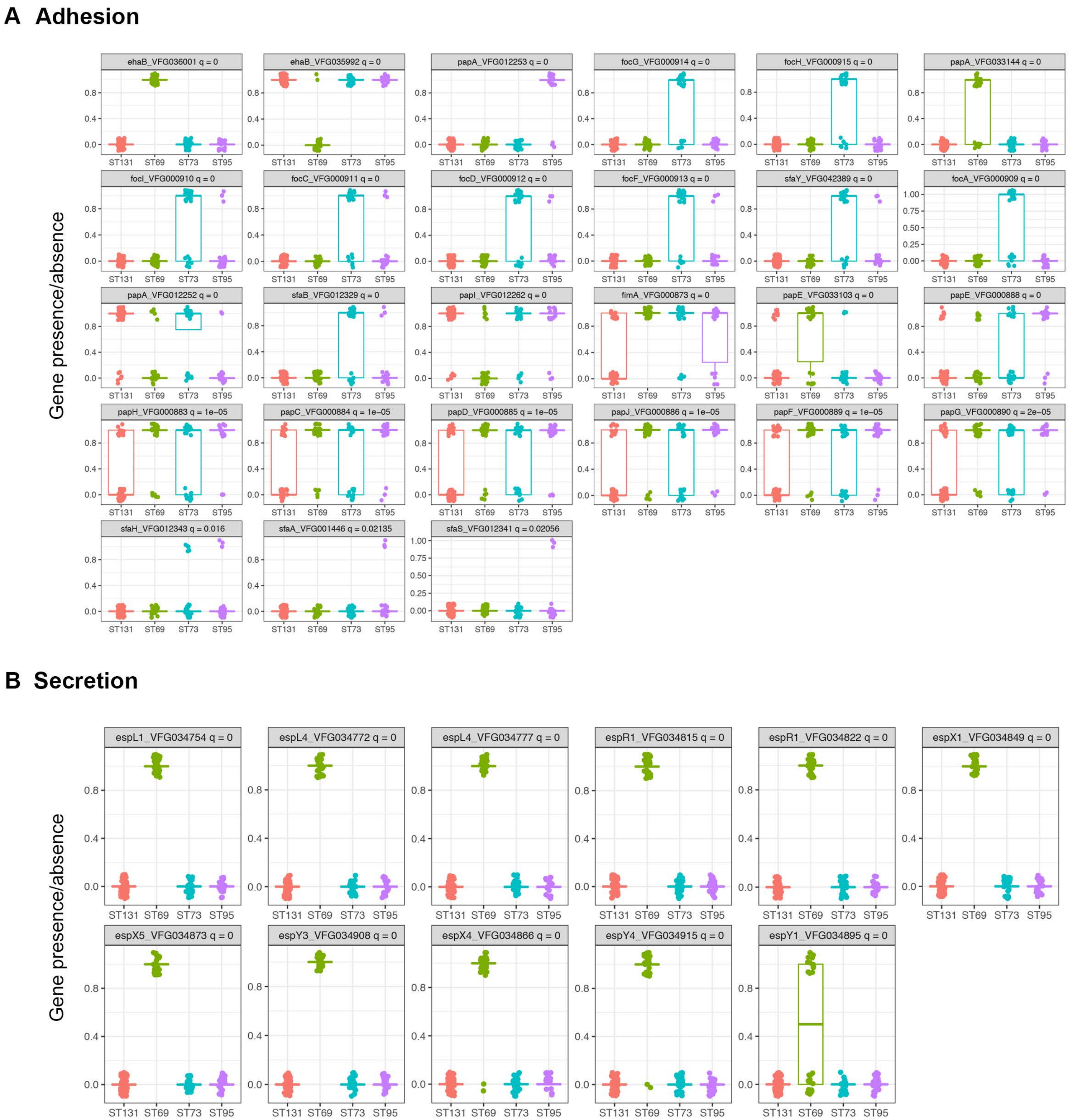

Figure S5. Individual occurrences of each indicated virulence gene within the different STs, grouped by function: A Adhesion, B, secretion . Genes are scored as present (1) or absent (0) with each circle representing a single isolate; symbols are dodged to aid in viewing. Calculated q values are shown in each graph header; values < 10-6 are assigned as 0.

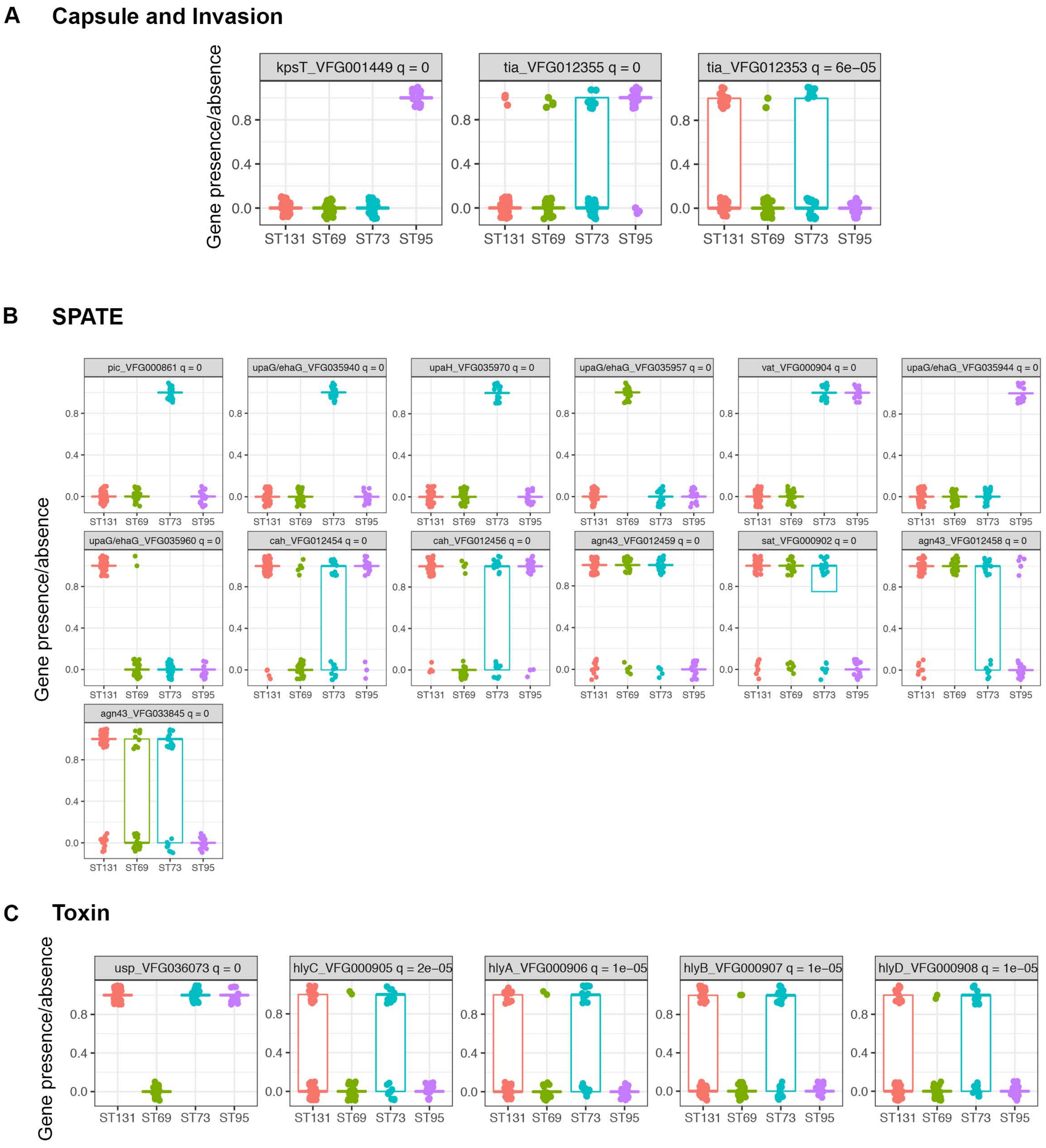

Figure S6. As figure S5, but grouped for A Capsule and invasion, B SPATE and C Toxin.

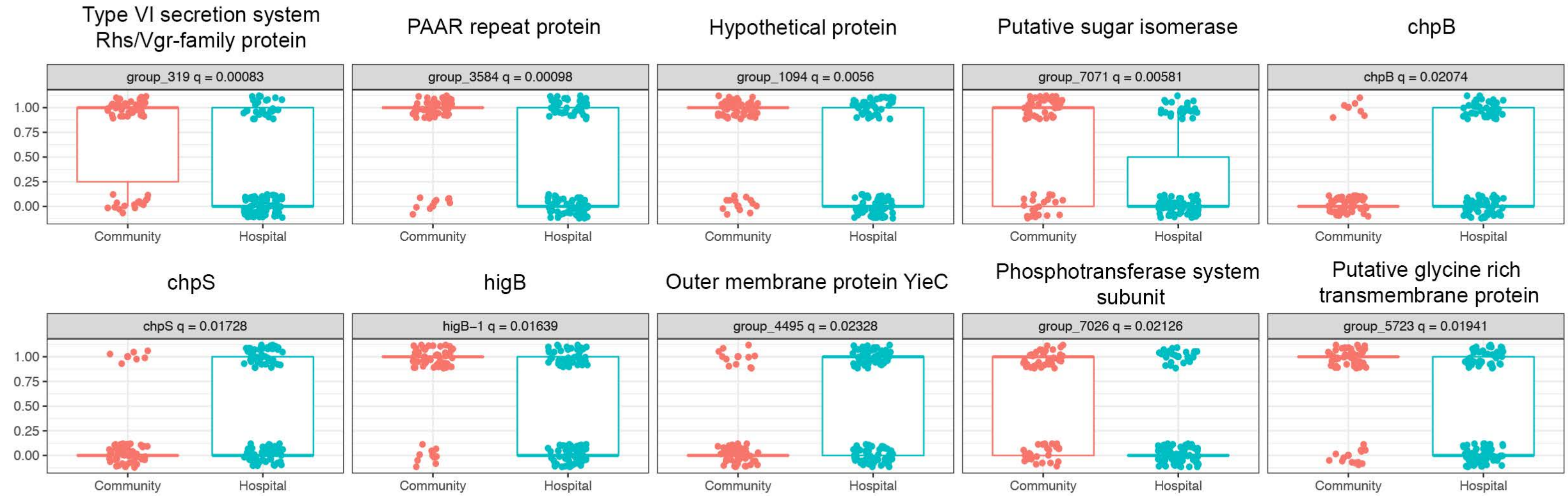

Figure S7. Individual occurrences of each indicated gene with significant corrected association between hospital and community-acquired infections, from the data summarised in Fig 7. Genes are scored as present (1) or absent (0) with each circle representing a single isolate; symbols are dodged to aid in viewing.

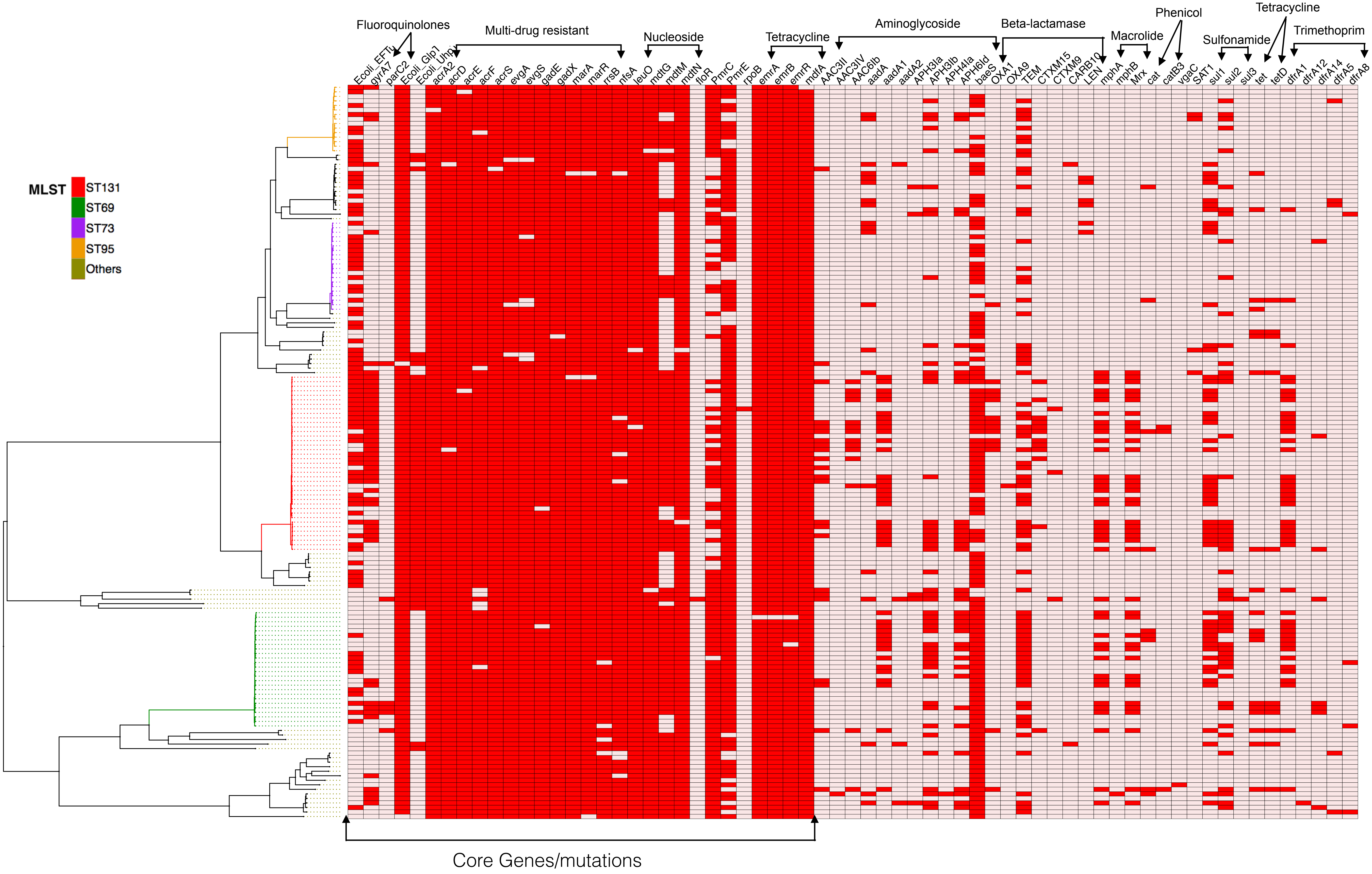

Figure S8. Antibiotic resistance genes found within the isolates. Presence (in dark red) of indicated antibiotic resistance genes within each isolate. STs are indicated in the dendrogram on the left of the figure, as described in Figure 1.
